# Endogenous Hormones and Antiretroviral Exposure in Plasma, Cervicovaginal Fluid, and Upper-Layer Packed Cells of Malawian Women Living with HIV

**DOI:** 10.1089/aid.2019.0278

**Published:** 2020-07-31

**Authors:** Melanie R. Nicol, Mackenzie L. Cottrell, Amanda H. Corbett, Lameck Chinula, Gerald Tegha, Frank Z. Stanczyk, Stacey Hurst, Athena P. Kourtis, Jennifer H. Tang

**Affiliations:** ^1^ Department of Experimental and Clinical Pharmacology, College of Pharmacy, University of Minnesota, Minneapolis, Minnesota, USA.; ^2^ Division of Pharmacotherapy and Experimental Therapeutics, School of Pharmacy, University of North Carolina at Chapel Hill, Chapel Hill, North Carolina, USA.; ^3^ Department of Obstetrics and Gynecology, School of Medicine, University of North Carolina at Chapel Hill, Chapel Hill, North Carolina, USA.; ^4^ UNC Project Malawi, Lilongwe, Malawi.; ^5^ Department of Obstetrics and Gynecology, Keck School of Medicine, University of Southern California, Los Angeles, California, USA.; ^6^ Division of Reproductive Health, Centers for Disease Control and Prevention, Atlanta, Georgia, USA.

**Keywords:** hormones, estradiol, antiretrovirals, efavirenz, tenofovir, adherence

## Abstract

Overlap in metabolism pathways of endogenous female sex hormones and antiretroviral drugs may lead to altered exposure to these compounds. In a family planning clinic in Lilongwe, Malawi, blood, blood cell, and cervicovaginal fluid (CVF) samples from seventy-three HIV positive Malawian women taken in follicular and luteal menstrual phases were assessed for estradiol and progesterone by chemiluminescent immunoassay, and for antiretroviral concentration by liquid chromatography-mass spectrometry. In both follicular and luteal phases, estradiol concentrations were lower in women receiving efavirenz compared with women on non-efavirenz regimens or no antiretroviral therapy (*p* < .01). Serum estradiol was moderately and negatively correlated with efavirenz plasma (*r* = −0.36, *p* < .001) and CVF (*r* = −0.50, *p* < .001) concentrations. Serum estradiol was a significant predictor of efavirenz CVF concentrations even after adjusting for efavirenz plasma concentrations (*p* = .02). In upper-layer packed cells (ULPCs), tenofovir diphosphate (TFVdp) concentrations were similar between follicular and luteal phases and were not correlated with estradiol or progesterone concentrations. Tenofovir concentrations in CVF were not associated with menstrual cycle or serum hormone concentrations. In CVF and plasma, efavirenz concentrations were negatively correlated with serum estradiol concentrations, suggesting a modulatory effect of estradiol on efavirenz metabolism and/or transport processes, and/or an effect of efavirenz on the metabolism of estradiol. Differences in CVF persisted even after adjusting for plasma concentrations, suggesting a mechanism specific to the female genital compartment separate from absorption or hepatic metabolism. In contrast, TFVdp concentrations in ULPC were not influenced by endogenous estradiol or progesterone concentrations.

## Introduction

Sex steroid hormones can modulate expression and activity of drug-metabolizing enzymes and transporters.^1,2^ Therefore, the disposition of antiretroviral drugs metabolized and/or transported by such pathways may be influenced by sex steroid hormones. Likewise, antiretroviral drugs may also influence the metabolism and disposition pathways of sex hormones. Globally, half of the people living with HIV are female.^3^ Therefore, it is critical to understand interactions between female sex hormones, such as estradiol and progesterone, and drugs used to treat HIV infection.

Differences in pharmacokinetics and efficacy of antiretroviral drugs between men and women have been observed.^4^ Similarly, pregnancy (which leads to high estradiol exposure) can significantly influence antiretroviral exposure.^5^ However, little information exists on menstrual cycle effects on variability in antiretroviral drug exposure that could affect efficacy or interpretation of adherence monitoring, whereas variability in the genital compartment could have implications for periodic viral shedding.

Here, we examined the relationship between serum estradiol and progesterone, across follicular and luteal phases of the menstrual cycle, and three antiretroviral drugs (tenofovir, efavirenz, and nevirapine) in three unique biological matrices: plasma, blood cells, and cervicovaginal fluid (CVF).

## Methods

### Study population

Nonpregnant women living with HIV aged 18–45 years were recruited from a family planning clinic in Lilongwe, Malawi. Enrollment details in this clinical trial (NCT03153709) have been previously published.^6^ This analysis includes data collected over the first two study visits, before randomization to hormonal contraceptive method. Women were eligible if they had at least two regular, monthly cycles in the 3 months preceding study enrollment and had not received hormonal or intrauterine contraception in the previous 6 months. Women with visible genital ulcers or lesions on pelvic exams were excluded, as were women with known or suspected genital tract cancer.

### Study visit and sampling

Eligible women underwent sampling over two visits, the first within 14 days from the onset of menses (follicular phase) and the second between 15 days from the onset of menses until the onset of next menses (luteal phase). Blood was centrifuged and separated into serum, from which estradiol and progesterone concentrations were measured using a competitive chemiluminescent immunoassay (Siemens Healthcare Diagnostics, Inc., Deerfield, IL). Lower limits of quantification were 20 pg/mL and 0.2 ng/mL for estradiol and progesterone, respectively. Visits were classified as follicular if visit 1 progesterone was <1.0 pg/mL and as luteal if visit 2 progesterone was ≥1.0 pg/mL.

An additional blood sample was collected for antiretroviral quantification. Women who took their antiretroviral drugs in the morning were asked to hold their dose until after sample collection so trough concentrations could be evaluated. The sample was centrifuged to separate plasma and upper-layer packed cell (ULPC) as previously described.^7^ Efavirenz and nevirapine were quantified in plasma and tenofovir diphosphate (TFVdp) in ULPC using validated liquid chromatography-mass spectrometry assays with lower limit of quantification (LLOQ) of 50 ng/mL, 1 ng/mL, and 10 pmol/mL, respectively. ULPC with plasma contamination were not analyzed.

To assess antiretroviral concentrations in genital compartment, CVF was collected from two Weck-Cel sponges by placing one sponge into the cervical os and the other into the right posterior vaginal fornix, each for 1 min to absorb secretions. Both sponges were placed into a single cryovial and stored at −80°C. Efavirenz, nevirapine, and tenofovir in Weck-cel sponges were quantified with a validated assay (LLOQ 0.26 ng/sample). Because the exact volume of CVF on each sponge is unknown, results are shown as ng per sample.

### Statistical analysis

Results are shown as median (25th–75th percentile) unless otherwise noted. Wilcoxon ranked sum test and logarithmic paired *t*-test were used to test for median concentration differences between menstrual phases and to compare hormone concentrations between antiretroviral regimens. Spearman rank sum test was used to test correlations. General linear models were used to test for differences in genital compartment antiretroviral concentrations after adjusting for plasma concentrations. Statistics were performed using SAS 9.4 (Cary, NC).

## Results

Samples were collected from 73 women with median age of 34 (range 23–44) years and median weight of 55 (interquartile range 50–63) kg. Samples were collected an average (median) of 15 hours postdose (range 13–22). Sixty-four women were on regimens including tenofovir disoproxil fumarate (300 mg daily) while only five were on zidovudine-containing regimens (300 mg twice daily). Fifty-eight were on efavirenz-containing regimens (600 mg daily), nine on nevirapine (200 mg twice daily), and two on boosted atazanavir (300 + 100 mg ritonavir daily). Four participants were not receiving antiretroviral therapy (ART) at time of sample collection.

### Estradiol and progesterone

**FIG. 1.**
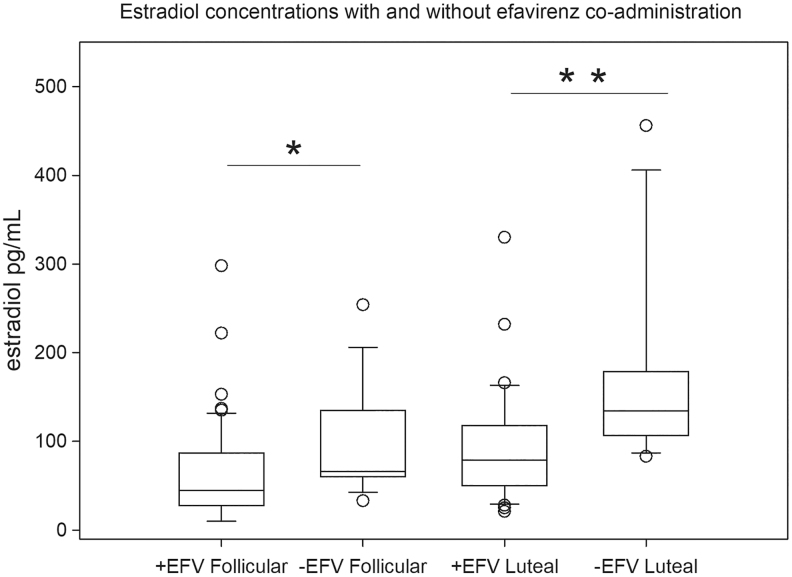
Estradiol concentrations with and without EFV co-administration. Estradiol concentrations in follicular and luteal phase were significantly lower in women receiving concomitant EFV. Available samples in each group by regimen (follicular: 55 on EFV; non-EFV group includes: 9 on nevirapine; 2 on atazanavir; 4 on no antiretroviral therapy. Luteal: 36 on EFV; non-EFV group includes: 7 on nevirapine; 2 on atazanavir; 3 on no antiretroviral therapy). *p < .01, **p < .001, Wilcoxon rank sum test. EFV, efavirenz.

Median serum estradiol concentrations were 57 (31–94) pg/mL in the follicular phase and 92 (60–134) pg/mL in luteal phase (*p* < .001). Estradiol concentrations significantly differed by antiretroviral regimen with lower estradiol observed in women receiving concomitant efavirenz (Fig. 1). There was no difference in estradiol concentrations between women on regimens containing atazanavir, nevirapine, or those on no-ART (*p* = .7), so these women were pooled for this comparison. Eight women, all receiving efavirenz, had estradiol below the assay limit (<20 pg/mL) in the follicular phase. Of these eight women, all six who also had a luteal phase sample collected had achieved an estradiol concentration above the assay limit in the luteal phase, and at least four had estradiol and progesterone concentrations consistent with recent ovulation (Supplementary Table S1).

By definition, all progesterone concentrations in the follicular phase were <1.0 ng/mL; 72% were below the assay lower limit of quantification (0.2 ng/mL), and therefore no correlations with progesterone were tested within follicular phase. Within the follicular phase, there were no significant differences in antiretroviral regimens between those above versus below the LLOQ. In luteal samples, median progesterone concentration was 4.7 (2.9–6.6) ng/mL.

### Efavirenz

**FIG. 2.**
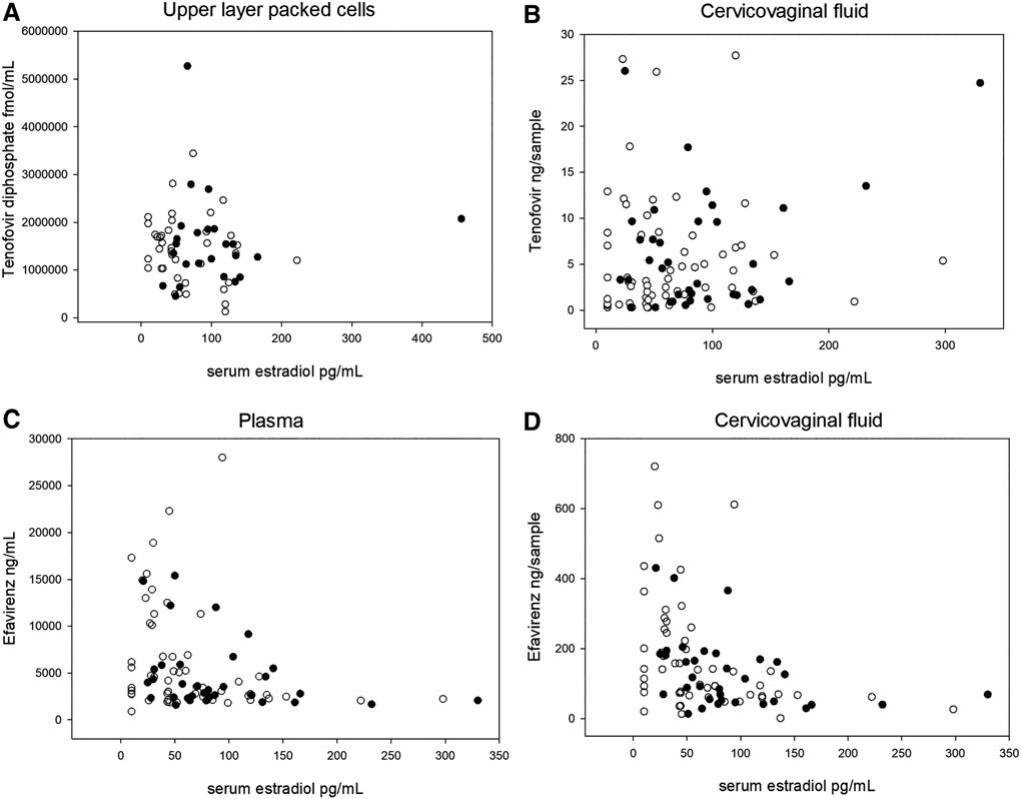
Correlations between antiretroviral drugs and serum estradiol concentrations. Serum estradiol concentrations are plotted between intracellular tenofovir diphosphate concentrations in upper layer packed cells **(A)**; tenofovir concentrations in cervicovaginal fluid **(B)**; efavirenz concentrations in plasma **(C)**; and efavirenz concentrations in cervicovaginal fluid **(D)**. *Open circles* represent samples collected in follicular phase; *filled circles* represent samples collected in the luteal phase. Concentrations in cervicovaginal fluid are expressed as ng drug per Weck-cel Sponge.

Efavirenz plasma and CVF concentrations are reported in Table 1. There was no significant difference between follicular and luteal phase in either plasma or genital compartment. When combining data from both menstrual phases, efavirenz plasma concentrations were moderately and negatively correlated with estradiol (*r* = −0.36; −0.54 to −0.16) but not significantly with progesterone concentrations (*r* = −0.19; −0.38 to 0.02). A negative correlation between estradiol and efavirenz plasma concentrations was observed within follicular phase (*r* = −0.35, −0.56 to −0.09) (Fig. 2C); in luteal phase the correlation did not reach significance (*r* = −0.27; −0.55 to 0.07, *p* = .21).

**Table 1.** Antiretroviral Drug Concentrations by Follicular and Luteal Phase in Plasma, Upper-Layer Packed Cells, and Cervicovaginal Fluid

	Follicular* n, *median (25th–75th percentile)	Luteal* n*, median (25th–75th percentile)	Wilcoxon rank sum, p-value	Logarithmic paired* t*-test* n, p*-value
TFVdp in ULPC, pmol/mL	41, 1,400 (1,030–1,800)	25, 1,520 (1,120–1,850)	.83	15, .56
Tenofovir in CVF, ng/samples	60, 3.28 (1.29–7.57)	38, 3.92 (1.63–9.64)	.50	35, .49
Efavirenz in plasma, ng/mL	54, 3,490 (2,490–6,910)	35, 3,220 (2,300–5,840)	.31	32, .97
Efavirenz in CVF, ng/sample	54, 123.9 (67–244.4)	35, 101.9 (48.6–180.7)	.28	32, .72
Nevirapine in plasma, ng/mL	9, 6,530 (5,540–9,010)	7, 5,440 (4,430–8,350)	.63	7, .65
Nevirapine in CVF, ng/sample	9, 286 (267.8–952.9)	7, 403 (131.3–495.3)	1.00	7, .48

CVF, cervicovaginal fluid; TFVdp, tenofovir diphosphate; ULPCs, upper-layer packed cells.

CVF efavirenz concentrations were highly correlated with plasma efavirenz concentrations (*r* = 0.78; 0.68–0.85). Similar to plasma, CVF efavirenz concentrations were also negatively correlated with estradiol overall (*r* = −0.50; −0.64 to −0.32) and within each menstrual phase (follicular *r* = −0.49; −0.66 to −0.25, luteal *r* = −0.38, *p* = .025) (Fig. 2D). Efavirenz CVF was also weakly negatively correlated with serum progesterone overall (*r* = −0.22; −0.41 to −0.01) and within luteal phase samples (*r* = −0.34, *p* = .047). Serum estradiol was a significant predictor of CVF efavirenz concentrations even after adjusting for efavirenz plasma concentrations (*p* = .02).

### Tenofovir

TFVdp concentrations in ULPC were similar between follicular and luteal phases (Table 1). There were no overall significant correlations between TFVdp concentrations and serum hormone concentrations. Similarly, TFVdp concentrations were not significantly correlated with estradiol in either follicular or luteal phase (Fig. 2A) nor with progesterone. Moreover, CVF tenofovir concentrations did not significantly differ by menstrual phase (Table 1) nor were they correlated with serum estradiol (Fig. 2B) or progesterone. ULPC TFVdp concentrations were not significantly correlated with CVF tenofovir concentrations.

### Nevirapine

Nevirapine concentrations did not significantly differ between phases in either plasma or CVF (Table 1). Correlations with nevirapine and serum hormones were not significant. Similar to efavirenz, nevirapine plasma concentrations were highly correlated with CVF nevirapine concentrations (*r* = 0.67; 0.24–0.87).

## Discussion

In CVF and plasma, efavirenz concentrations were negatively correlated with serum estradiol concentrations, suggesting either a modulatory effect of estradiol on efavirenz metabolism and/or transport processes, and/or a potential two-way interaction with efavirenz modulating estradiol metabolism. In contrast, tenofovir-diphosphate concentrations in ULPCs did not appear to be associated with endogenous estradiol or progesterone concentrations. Antiretroviral concentrations did not significantly differ by menstrual phase, possibly due to overlap of estradiol concentrations between phases.

Efavirenz's primary elimination pathway is hepatic metabolism by the cytochrome p450 system, with CYP2B6 being the predominant contributor.^8^ In hepatocytes extracted from human female livers, estradiol was shown to significantly induce CYP2B6 messenger RNA (mRNA) expression and activity.^9^ Increased CYP2B6 (or other hepatic enzyme) activity would explain lower systemic efavirenz exposure with increasing estradiol concentrations. Similarly, efavirenz is a substrate for efflux drug transporter MRP4,^10,11^ which like CYP2B6 may also be induced by estradiol. Increased expression of efflux drug transporter in the gut epithelium may point to decreased efavirenz absorption and is consistent with our findings of lower efavirenz concentrations with increased estradiol.

The negative correlation between estradiol and efavirenz concentrations could also be due to efavirenz inducing metabolism of estradiol. Efavirenz is also a known inducer of CYP-mediated metabolism, and several CYP enzymes are responsible for hydroxylation of estradiol to its metabolites.^8^ We found significantly lower estradiol concentrations in women receiving efavirenz compared with women receiving atazanavir- or nevirapine-based regimens, or who had not yet started ART. On average, exposure was 30%–40% lower in efavirenz users in both follicular and luteal phases. Efavirenz modulation of estradiol exposure has previously been reported in a randomized trial of pregnant Ugandan women where estradiol concentrations decreased significantly in women randomized to efavirenz treatment compared with women randomized to treatment with a protease inhibitor.^12^

Although other antiretrovirals could also impact estradiol metabolism through inhibition (protease inhibitors) or induction (nevirapine) of estradiol metabolism, we did not observe significant differences between users of these regimens and individuals on no ART, although our sample size for these groups was small. Estradiol supports the maintenance of a healthy pregnancy; in this study lower concentrations were correlated with lower birth weight and increased frequency of small for gestational age births. Outside of impacting pregnancy outcomes, prolonged low estradiol may lead to other clinical sequelae, such as decreased bone mineral density and hot flashes. However, in our study, the low estradiol concentrations were found only during the follicular phase (when estradiol concentrations are lowest) and at a single time point, and most of these women had subsequent elevations of their estradiol and/or evidence of ovulation, so it is unlikely that they had prolonged low estradiol exposure.

Serum estradiol remained a significant predictor of efavirenz concentration in CVF even after adjusting for plasma concentrations, suggesting a mechanism specific to the female genital compartment separate from absorption or hepatic metabolism. CYP2B6 protein is highly expressed in cervicovaginal tissue and compartment-specific CYP-mediated metabolism has been observed for some compounds.^13^ MRP4 protein and mRNA is highly expressed in vaginal epithelium, with decreased mRNA expression in tissues from postmenopausal women (decreased estrogen).^14^ This may lead to estradiol-induced increased efflux from the vaginal epithelium. Together, these data suggest a differential influence of estradiol on these metabolism and transport processes between tissue compartments and highlights the importance of studying tissue-specific processes directly.

Measurement of TFVdp in ULPC is one method to monitor antiretroviral adherence that provides an advantage over plasma in assessing longer term adherence due to the long half-life of TFVdp in red blood cells (RBCs) and peripheral blood mononuclear cells (PBMCs) but, unlike PBMC isolation, is simple to process, even in resource-limited settings. It is also plausible that female sex hormones modulate expression and activity of kinases and 5′ nucleotidases responsible for phosphorylation and dephosphorylation of TFVdp, as has been demonstrated *in vitro*.^15^ Previous studies have found sex differences in TFVdp concentrations in PBMC and in RBC,^16^ although the mechanism is unknown and could be related to differences in volume of distribution between sexes. Whether variable exposure to estradiol throughout the menstrual cycle impacts TFVdp could influence interpretation of drug concentrations related to adherence, and potentially compromising drug efficacy. We found, however, no significant variation in TFVdp ULPC concentrations, or tenofovir CVF concentrations, by menstrual phase, or by estradiol or progesterone concentrations. This finding may support the fact that sex differences in tenofovir disposition are due to factors other than hormonal regulation. It is also possible, due to the long half-life and retention of TFVdp in these cells, any mechanistic regulatory effect of sex hormones on TFVdp in ULPC is not reflected in the cyclic changes of serum estradiol across the menstrual cycle. In other words, since TFVdp in ULPC reflects average exposure over the past weeks, any fluctuations due to estradiol regulation are not observed. The lack of effect on tenofovir in CVF could reflect cell populations in the genital tract with differential responsiveness to estradiol regulation. Although differential benchmarks for adherence have been suggested between men and women, our data reassure that fluctuations in hormones throughout menstrual cycle do not need to be accounted for when ULPC are used for adherence monitoring of tenofovir.

There are limitations to this analysis. Importantly, this was a sub-analysis of a larger trial and patients were not randomized to their antiretroviral regimen. Our ability to detect significant differences in antiretroviral concentrations between menstrual phases was likely limited due to sample size; however, a *post hoc* analysis suggests our sample size would have been able to detect a 55% difference in efavirenz concentrations between follicular and luteal phases. In addition, the low number of participants on non-efavirenz containing regimens required these individuals to be pooled and therefore precludes any determinations on the effects of other medications on estradiol exposure. Lastly, although we identified significant correlations between estradiol and efavirenz, the association was highly variable, likely due to unaccounted for factors contributing to interindividual variability in estradiol and/or efavirenz exposure.

In conclusion, efavirenz concentrations were negatively correlated with serum estradiol concentrations, suggesting a modulatory effect of estradiol on efavirenz metabolism and/or transport processes, and/or an effect of efavirenz on the metabolism of estradiol. Differences in CVF efavirenz concentrations persisted even after adjusting for plasma concentrations, suggesting a mechanism specific to the genital compartment separate from absorption or hepatic metabolism that should be further explored to understand implications in viral shedding. In contrast, TFVdp concentrations in ULPC, which is used as a marker of adherence to tenofovir, were not influenced by the physiologic fluctuations in endogenous estradiol or progesterone concentrations.

## Supplementary Material

Supplemental data
