# Supplementary material for: Endogenous Hormones and Antiretroviral Exposure in Plasma, Cervicovaginal Fluid, and Upper-Layer Packed Cells of Malawian Women Living with HIV
Source: AIDS Res Hum Retroviruses. 2020 Jul 31;36(8):641–6. doi: 10.1089/aid.2019.0278 (PMC7414802; doi:10.1089/aid.2019.0278)
Supplement: Supplemental data [file Supp_TableS1.pdf]

Supplementary Data

Supplementary Table S1. Cases with Estradiol <20 pg/mL

		Follicular		Luteal	
Age (years)	*Antiretroviral regimen*	*Estradiol, pg/mL*[a]	*Progesterone, ng/mL*[b]	*Estradiol, pg/mL*[a]	*Progesterone, ng/mL*[b]
29	EFV/TDF/3TC	<20	<0.2	N/A	N/A
44	EFV/TDF/3TC	<20	<0.2	30	6.6
39	EFV/TDF/3TC	<20	<0.2	120	0.3[c]
33	EFV/TDF/3TC	<20	<0.2	81	6.5
30	EFV/TDF/3TC	<20	0.3	26	0.2[c]
28	EFV/TDF/3TC	<20	0.6	35	1.0[c]
29	EFV/TDF/3TC	<20	<0.2	95	5.8
27	EFV/TDF/3TC	<20	0.3	N/A	N/A

[a]Assay limit of quantification = 20 pg/mL.
[b]Assay limit of quantification = 0.2 ng/mL.
[c]These ''luteal'' samples were excluded from overall analysis since progesterone did not reach >1.5 ng/mL (see Methods section in the main text) but are included here to provide complete picture of those women with estradiol <20 ng/mL at follicular stage.

3TC, lamivudine; EFV, efavirenz; N/A, samples were not collected or were unevaluable; TDF, tenofovir disoproxil fumarate.
